# Supplementary material for: The causal relationship between the human gut microbiota and pyogenic arthritis: a Mendelian randomization study
Source: Front Cell Infect Microbiol. 2024 Nov 26;14:1452480. doi: 10.3389/fcimb.2024.1452480 (PMC11629706; doi:10.3389/fcimb.2024.1452480)
Supplement: Supplementary file 1 [file DataSheet1.zip › mendelian test/ebi-a-GCST90027583.csv_leave_one_out.pdf]

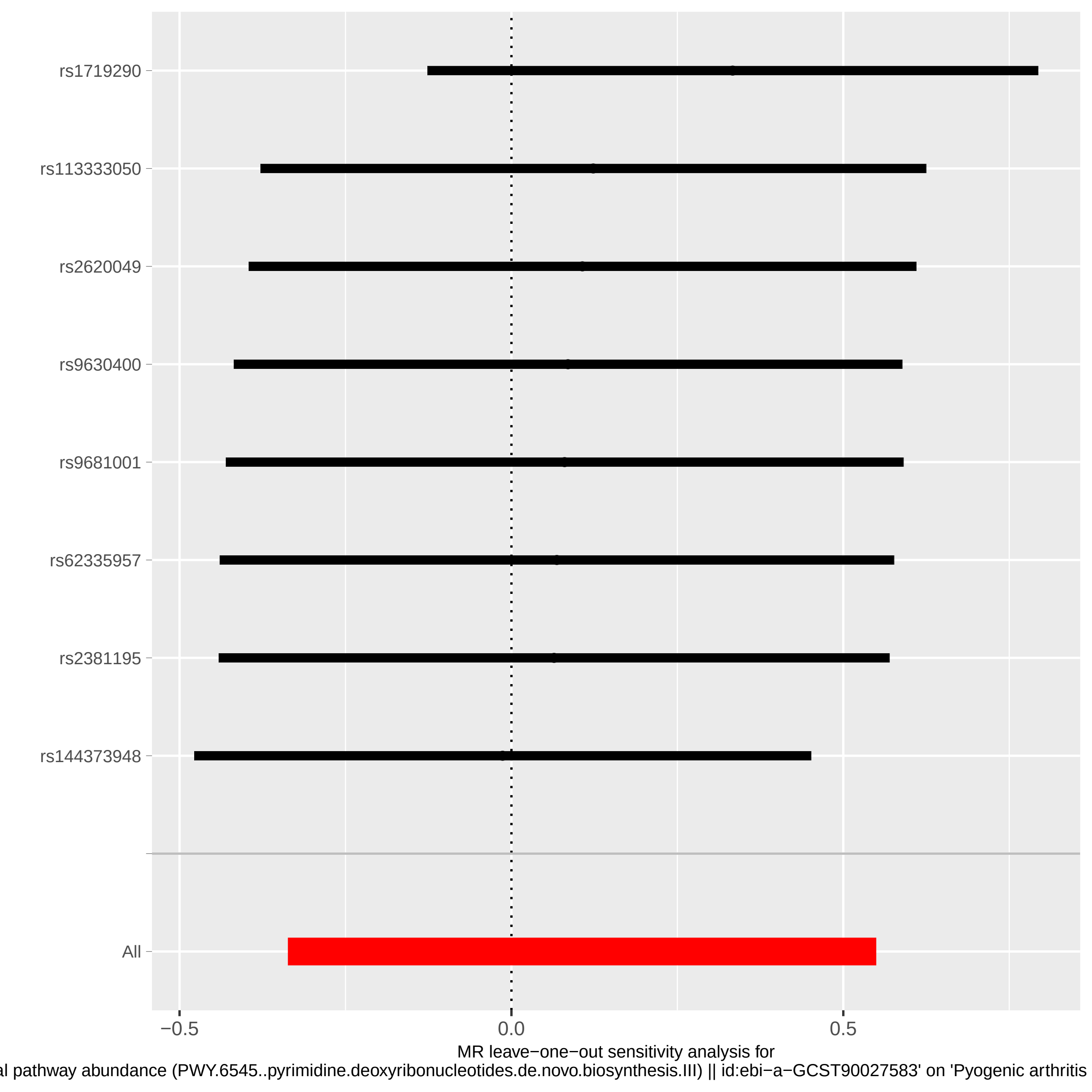
rs1719290
rs113333050
rs2620049
rs9630400
rs9681001
rs62335957
rs2381195
rs144373948
All
−0.5
0.0
0.5
MR leave−one−out sensitivity analysis for
al pathway abundance (PWY.6545..pyrimidine.deoxyribonucleotides.de.novo.biosynthesis.III) || id:ebi−a−GCST90027583' on 'Pyogenic arthritis
